# Postoperative change in patellofemoral alignment following closing-wedge distal femoral osteotomy performed for valgus osteoarthritic knees

**DOI:** 10.1186/s43019-020-00035-6

**Published:** 2020-03-16

**Authors:** Yusuke Akaoka, Hiroshi Nakayama, Tomoya Iseki, Ryo Kanto, Keiji Tensho, Shinichi Yoshiya

**Affiliations:** 1 Department of Orthopedic Surgery, Hyogo College of Medicine, 1-1 Mukogawa-cho, Nishinomiya, Hyogo 653-8501 Japan; 2 Department of Orthopedic Surgery, Shinshu University School of Medicine, Matsumoto, Nagano Japan

**Keywords:** Medial closed distal femoral osteotomy (DFO), Valgus deformity, Osteoarthritis of the knee, Patellar height, Patellofemoral joint congruity

## Abstract

**Purpose:**

To evaluate the postoperative change in patellar position after medial closed distal femoral osteotomy (DFO) performed for valgus osteoarthritic knees.

**Methods:**

The study included 21 consecutive knees in 20 patients undergoing DFO. A minimum of 2-year follow-up data was obtained for all subjects with a mean follow-up period of 42 months (range 31–59 months). The patellar position was evaluated on plain radiographs preoperatively, 1-year postoperatively, and 2-year postoperatively. For patellar height, the modified Insall–Salvati Index (mISI), modified Caton–Deschamps Index (mCDI) and modified Blackburne–Peel Index (mBPI) were measured on the standing lateral radiographs. Patellofemoral alignment on the axial plane was assessed on skyline views with 30° flexion based on the measurements for lateral patellar tilt (LPT) and lateral patellar shift (LPS). Measured values at pre- and postoperative phases were statistically compared using a two-way analysis of variance.

**Results:**

All indices including mISI, mCDI, mBPI, LPT and LPS showed no statistically significant postoperative changes.

**Conclusion:**

Medial closed-wedge DFO performed for valgus osteoarthritic knees did not significantly influence patellofemoral alignment either on the sagittal or axial plane. Therefore, to highlight the clinical relevance of our findings, medial closed-wedge DFO for the valgus knee does not adversely affect the patellofemoral joint.

**Level of evidence:**

Level IV, case series.

## Introduction

Osteotomy around the knee is adopted as a primary surgical option for knees with unicompartmental osteoarthritis in patients with high activity levels. In recent years, medial opening-wedge high tibial osteotomy has been widely used for knees with varus deformity, and good treatment outcomes have been reported in the literature [1–3]. On the other hand, there have also been reports that describe its effects on the patellofemoral joint as a result of patella baja and arthritis [4–10].

For lateral osteoarthritis in young patients or highly active patients with valgus deformity, the cause of the deformity is often located at the distal end of the femur and in the valgus position. We therefore performed a distal femoral osteotomy (DFO) for deformity correction. There are two methods of operation for DFO, including the medial closed-wedge method and the lateral open-wedge method. There are advantages and disadvantages for each method, but long-term results of more than 10 years have been reported for medial closed DFO [11, 12]. However, there are no detailed reports on the changes in the patellar position and effects on the patellofemoral joints that result from medial closed DFO.

The purpose of this study, therefore, is to investigate and evaluate the effects of medial closed DFO on the patellar position when performed for valgus knees with lateral compartment osteoarthritis.

## Materials and methods

### Patients

This was a retrospective study. In total, 21 consecutive knees in 20 patients (11 men and nine women) undergoing DFO from July 2013 to March 2016 comprised the study population. The mean age of the patients at surgery was 46.4 years, ranging from 29 to 68 years. All patients were tracked for a minimum of 2 years, and the mean follow-up period was 42 months (range 31 to 59 months) (Table 1).

**Table 1:** Patient demographics

Variable	
Number of patients/knees	20/21
Sex (male/female)	11/9
Age (years)	46.4 ± 12.9
Body mass index (kg/m^2^)	23.1 ± 3.0

Results are shown as mean ± standard deviation or *n*/*n*

The design of this study was approved by our Institutional Ethics Review Board and informed consent was obtained from all patients.

### Radiographic assessments

**Fig. 1:**
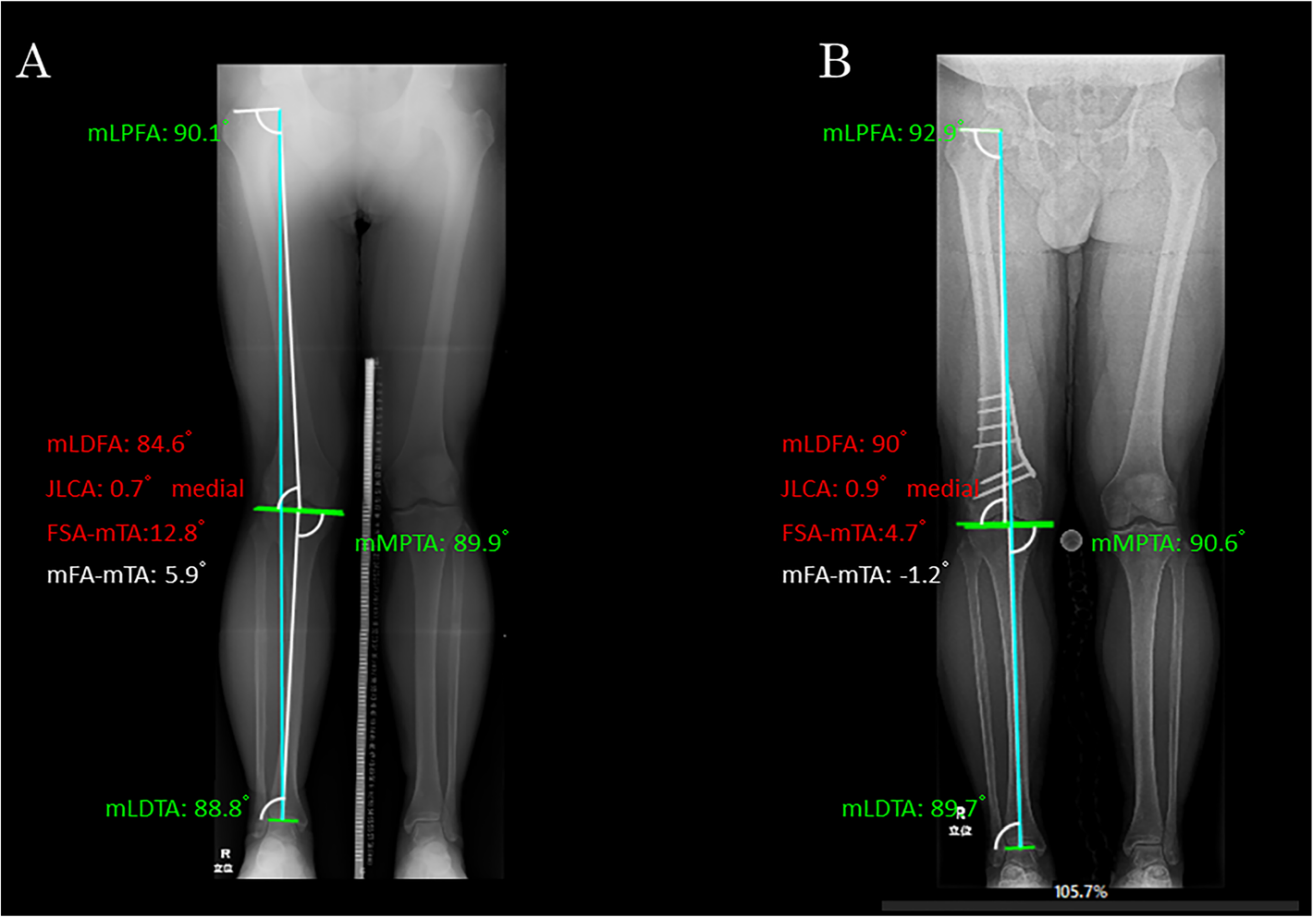
Measurement of lower extremity alignment on the long-leg weight-bearing radiograph using the image analysis software (mediCADR). **a** Preoperative and (**b**) Postoperative. Blue line (mechanical axis). white line (mechanical femoral and tibial axis). FSA femoral shift axis, JLCA joint line conversion angle, mFA mechanical femoral angle, mLDFA mechanical lateral distal femoral angle, mLDTA mechanical lateral distal tibial angle, mLPFA mechanical lateral proximal femoral angle, mMPTA mechanical medial proximal tibial angle, mTA mechanical tibial axis

The alignment parameters were measured on the long-leg weight-bearing radiograph. The parameters subjected to the analysis were as follows: mechanical lateral distal femoral angle (mLDFA), mechanical medial proximal tibial angle (mMPTA) and mechanical tibiofemoral angle (mTFA). The radiological measurements were performed using digital planning software (mediCADR, Hectec, Germany) [13]. The assessments were performed before surgery as well as at 1 and 2 years after surgery. Subsequently, the measured values at each of the time periods were compared (Fig. 1).

**Fig. 2:**
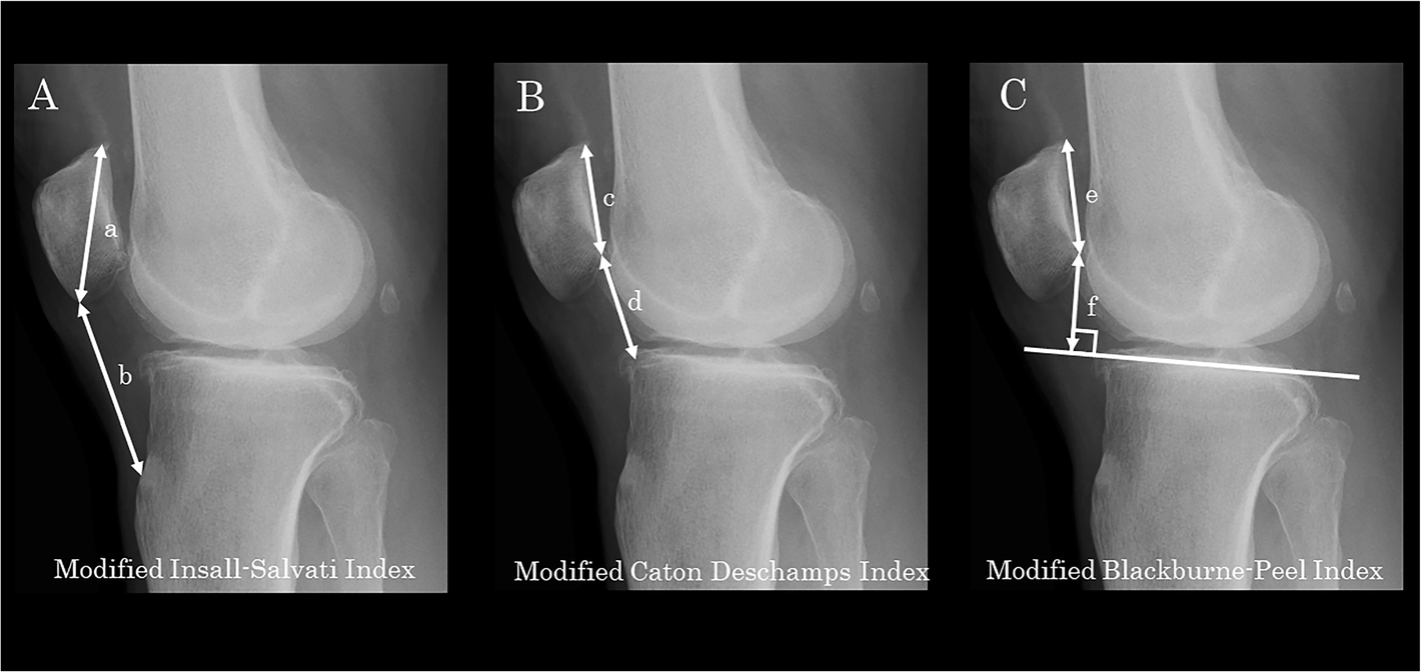
Measurements of patellar height on a standing lateral radiograph. **A** Modified Insall–Salvati index (b/a), defined as the value obtained by dividing the patellar tendon length (b, from inferior pole of the patella to the distal insertion of the patellar tendon at the tibial tubercle) by the maximum length of the diagonal length of the patella (a). **B** Modified Caton–Deschamps index (d/c), defined as the value obtained by dividing the distance from the distal end of the articular surface of the patella to the angular protrusion of the tibial plateau (d) by the length of the patellar articular surface (c). **C** Modified Blackburne–Peel index (f/e), defined as the value obtained by dividing the length of the perpendicular line from the lowest pole of the patellar articular surface to the surface of the tibial plateau (f) by the length of the patellar articular surface (e)

The patellar height on the sagittal plane was assessed on the weight-bearing lateral radiograph in an extended position. The patellar height was measured on the standing lateral radiograph in an extended position. In their original descriptions, the Insall–Salvati [14], Caton–Deschamps [15], and Blackburne–Peel [16] indices were based on the measurement of the nonweight-bearing lateral radiograph in mild degrees of flexion (30°). The assessment in an extended position in this study may have been associated with reduced tension in the extensor mechanism and slackness of the patellar tendon leading to potential inaccuracy in assessment of the patellar height. As for the analytical parameters, the modified Insall–Salvati Index (mISI), modified Caton–Deschamps Index (mCDI), and modified Blackburne–Peel Index (mBPI) [6] were measured (Fig. 2).

**Fig. 3:**
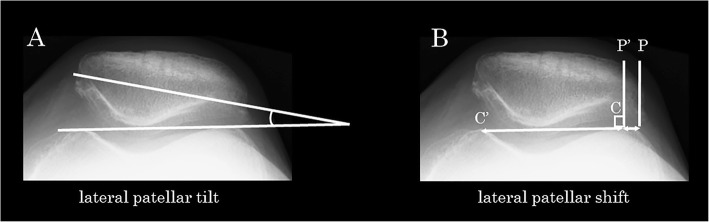
Assessment of patellofemoral alignment on a skyline view radiograph with the knee in 30° of flexion. **a** Lateral patellar tilt, defined as the angle formed by the line passing through the maximum width of the patella and the tangent to the anterior apices of the medial/lateral femoral condyles. **b** Lateral patellar shift (P–P’/C–C’), defined as the value obtained by dividing the distance from the perpendicular line of the patellar lateral margin to the apex of the lateral femoral condyle (P–P’) by the distance from apices of the medial and lateral femoral condyles (C–C’) with tangent to the anterior apices of the medial/lateral femoral condyles as the baseline

Regarding the patellofemoral joint alignment on the axial plane [17], the lateral patellar tilt (LPT) and lateral patellar shift (LPS) were measured on the skyline view radiograph with the knee in 30° of flexion (Fig. 3). All measurements were repeated twice by a single certified orthopedic surgeon (YA), and the mean values were subsequently calculated.

Excellent reliability of radiological measurement with the computer software used in the analysis of this study has been confirmed in a previous study [13].

### Surgical procedures

All surgeries were performed by a single surgeon (HN) with the patient under general anesthesia.

#### Arthroscopic procedure

Arthroscopic evaluation was conducted prior to the osteotomy. For the intra-articular arthroscopic procedure, all osteophytes in the accessible area were removed. Ten of 21 knees underwent lateral meniscus reconstruction using an autogenous semitendinosus tendon graft. One knee underwent lateral meniscal suture repair, while partial lateral meniscectomy was performed in three knees. Moreover, one knee with restricted patellar mobility underwent arthroscopic lateral retinacular release.

#### Osteotomy technique

DFO was performed using a biplanar medial closed-wedge technique [18–20]. A 4- to 5-cm longitudinal skin incision was made on the medial side of the distal femur. The fascia of the vastus medialis was excised, and the vastus medialis was elevated to expose the medial femur. A hinge was left intact at the lateral part of the osteotomy site, and a biplanar osteotomy was performed. A TomoFix medial distal femur anatomical plate (DePuy Synthes, Solothurn, Switzerland) was used for a minimally invasive plate osteosynthesis technique. After the osteotomy and fixation were completed, a surgical drain was placed and the wound was closed.

#### Postoperative management

When DFO was performed alone without concomitant meniscal reconstruction/repair procedures, the operated knee was not immobilized, and a range of motion exercises was started at several days after surgery. In the knees that underwent concomitant meniscal reconstruction or repair, the operated knee was immobilized in extension for 1 week and a range of motion exercises was started thereafter. Partial weight-bearing (20 kg) was initiated at 3 weeks postoperatively, and full weight-bearing was allowed at 4 weeks.

### Statistical analysis

The intrarater reliability was determined using intraclass correlation coefficients. Postoperative sequential changes through preoperative, 1-year postoperative, and 2-year postoperative periods were statistically assessed using a two-way analysis of variance. Statistical comparison of pre- and postoperative coronal alignment parameters of the lower extremity was performed using a paired *t* test. We defined statistical significance as *P* < 0.05. A minimum sample size of nine knees was required for an α value of 0.05 and β value of 0.8. Based on past literature, a clinical difference of 0.2 for the mISI and standard deviation of 0.15 were considered [5]. All analyses were performed with commercially available software (Stat Flex version 5.0, Artech, Inc., Osaka, Japan).

## Results

The intrarater reliability was shown to be excellent (intraclass correlation coefficient = 0.872). The pre- and postoperative radiological parameters measured for coronal alignment of the lower extremity were as follows: mLDFA, from 84.3 ± 2.0° to 89.3 ± 1.8° with significant postoperative increase (*P* < 0.001); mTFA, from 4.4 ± 4.7° valgus to 0.2 ± 4.7° varus with significant postoperative change (*P* < 0.001) (Table 2). The preoperative, 1-year postoperative and 2-year postoperative parameters related to the patellar height were as follows: 1.1 ± 0.2, 1.1 ± 0.2 and 1.1 ± 0.2 for mISI; 1.0 ± 0.2, 1.0 ± 0.2 and 0.9 ± 0.1 for mCDI; and 0.9 ± 0.2, 0.8 ± 0.2 and 0.8 ± 0.2 for mBPI, respectively (not significant). No significant changes over time were seen in all parameters (Table 3). The preoperative, 1-year postoperative and 2-year postoperative radiological parameters related to the patellofemoral alignment were 5.1 ± 3.9°, 4.6 ± 4.2° and 4.4 ± 4.4° for LPT, and 6.8 ± 3.4%, 6.8 ± 6.0% and 7.7 ± 4.4% for the LPS, respectively (not significant). Again, both parameters showed no significant changes over time (Table 4). When the LPT and LPS were examined for individual cases, 13 out of 21 cases were found to have decreased tilt after surgery, four cases had no change, and four cases had an increase in tilt. Nine out of 21 cases had a decrease in postoperative shift, two had no change, and 10 had an increase in shift.

**Table 2:** Knee alignment parameter for indices before and after distal femoral osteotomy

	Preoperative	Postoperative	*P* value
mLDFA (°, surgery side)			
Average (SD)	84.3 (2.0)	89.3 (1.8)	<0.001
Range	79.5–86.6	87.0–94.0	–
Median	84.7	89.3	–
mLDFA (°, nonsurgery side)			
Average (SD)	86.0 (1.6)	–	–
Range	83.1–89.1		
Median	86.1		
mMPTA (°)			
Average (SD)	86.5 (2.3)	–	–
Range	82.0–89.5		
Median	86.9		
mTFA (°)			
Average (SD)	4.4 (4.7)	−0.2 (4.0)	< 0.001
Range	−1.0 to 14.6	−8.5 to 8.0	–
Median	3.0	0	–
Femoral closed distance (mm)		Intraoperative	
Average (SD)		5.5 (1.7)	
Range		3.0–9.0	
Median		5.5	

Values less than 0.05 were considered significant

*mLDFA* mechanical lateral distal femoral angle, *mMPTA* mechanical medial proximal tibial angle, *mTFA* mechanical tibiofemoral angle, *SD* standard deviation

**Table 3:** Results of patellar height measurement

	Preoperative	1 year postoperative	2 years postoperative
Modified Insall–Salvati Index			
Average (SD)	1.1 (0.2)	1.1 (0.2)	1.1 (0.2)
Range	0.7–1.4	0.7–1.4	0.70–1.4
Modified Caton–Deschamps Index			
Average (SD)	1.0 (0.2)	1.0 (0.2)	0.9 (0.1)
Range	0.8–1.4	0.7–1.3	0.6–1.2
Modified Blackburne–Peel Index			
Average (SD)	0.9 (0.2)	0.8 (0.2)	0.8 (0.2)
Range	0.4–1.2	0.3–1.2	0.28–1.1

*SD* standard deviation

**Table 4:** Results of patellofemoral joint congruity

	Preoperative	1 year postoperative	2 years postoperative
Lateral patellar tilt (°)			
Average (SD)	5.1 (3.9)	4.6 (4.2)	4.4 (4.4)
Range	1.0–14.0	−2.0 to 14.0	−1.0 to 14.0
Lateral patellar shift (%)			
Average (SD)	6.8 (3.4)	6.8 (6.0)	7.7 (4.4)
Range	−2.0 to 14.0	−7.0 to 24.0	−1.0 to 19.0

*SD* standard deviation

## Discussion

The most important finding of the present study was that there were no significant changes in the patellar height and patellofemoral joint congruity after medial closed DFO on valgus osteoarthritic knees for up to 2 years postoperatively.

There have been a number of studies examining the effect of opening-wedge high tibial osteotomy on patellar position and patellofemoral alignment. The majority of those studies have shown postoperative reduction of patellar height (patella baja). Previously reported causative factors for postoperative patella baja are distal translation of the tibial tubercle associated with wedge opening [8], postoperative shortening of the patellar tendon due to peritendinous fibrosis secondary to surgical invasion [21] and increased posterior tibial slope after osteotomy [22]. In DFO, osteotomy is made proximally to the patellofemoral joint without changes in the geometry of the proximal tibia or surgical intervention around the patellar tendon. Consequently, it is assumed that the DFO procedure induces less change for the patellofemoral alignment compared to opening-wedge high tibial osteotomy.

To the best of our knowledge, there have been no studies that investigate the change in patellar height following DFO performed for valgus osteoarthritic knees. In terms of DFO performed to correct valgus deformity in knees with patellar instability, several studies have examined the postoperative change in patellar height; however, the results have been inconsistent between studies. Nha et al. [23] reported that there was no significant postoperative change in patellar height as assessed by the Caton–Deschamps index in knees that underwent medial closed DFO for patellar dislocation associated with valgus knee deformity. On the other hand, Wilson et al. [24] reported a significant reduction in the Caton–Deschamps index following lateral open DFO performed for recurrent patellar instability. Participants in the present study had valgus osteoarthritic knees that underwent medial closed DFO, and no change in patellar height was induced by the osteotomy procedure in this study population.

**Fig. 4:**
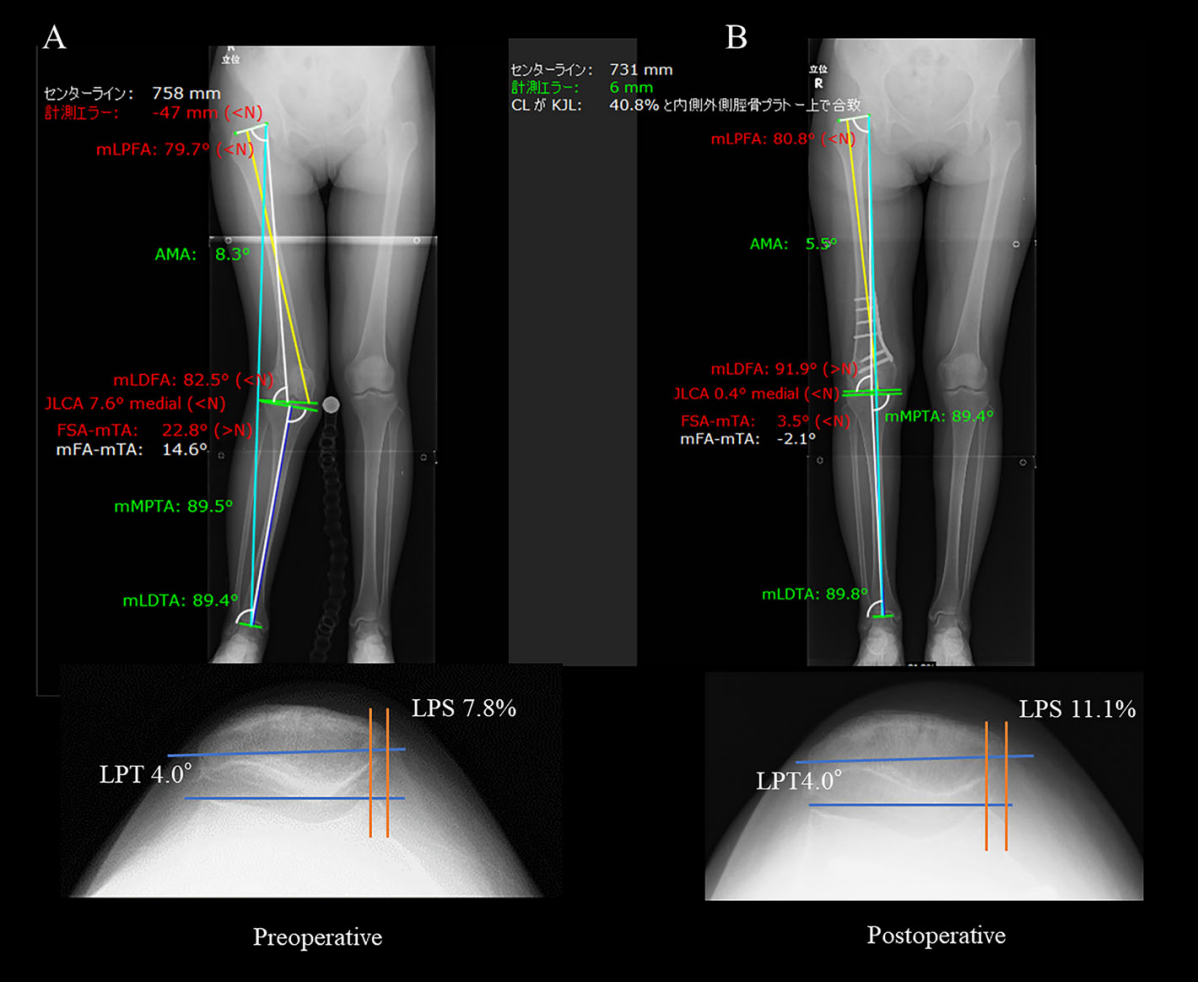
Representative case of changes in total leg length and patellar congruity before and after surgery. **a** Preoperative in a 53-year-old female; the patient is presented with a valgus knee with mechanical lateral distal femoral angle (mLDFA) 82.5° and mechanical femoral angle (mFA)-mechanical tibial angle (mTA) 14.6°. Lateral patellar tilt (LPT) was 4.0° and lateral patellar shift (LPS) was 7.8%. **b** Postoperative; the mLDFA and mFA-mTA changed to 91.9° and -2.1°, respectively. LPT remained unchanged at 4.0° and the LPS was 11.1%. FSA femoral shift axis, JLCA joint line conversion angle, mLDTA mechanical lateral distal tibial angle, mLPFA mechanical lateral proximal femoral angle, mMPTA mechanical medial proximal tibial angle

In terms of the tilt and shift of the patella, there are some reports that suggest that DFO for patellar instability with valgus deformity of the knee may lead to significant improvement [23, 25–27]. This is because the valgus moment to the patella is reduced by decreasing the Q angle, and the congruity of the patella is thus improved. However, these results deviate from our study, which showed that there is no effect on the patellar tilt or shift because combined procedures were proactively performed with DFO for patellar instability to improve patellar tracking, including tibial tuberosity osteotomy [23, 26], medial reefing [23, 25, 26] and lateral release [23, 26, 27]. In our study, the mean preoperative tilt and shift were small at 5.1° and 6.8%, respectively, and there were no cases with patellar instability. One case showed poor patellar mobility and underwent lateral release; however, all cases that were included in this study exhibited good patellofemoral joint congruity compared to preoperative congruity. Although there were cases that showed some degree of improvement in preoperative tilt and shift, these cases showed little pre- to postoperative change. Moreover, even for cases with great improvement in the Q angle (mTFA), there were no change in the patellar congruity (Fig. 4), and we believe that DFO does not affect the patellar tilt or shift. We believe that these results are consistent with our study that shows that medial closed DFO for valgus deformity does not affect the height, tilt or shift of the patella in patients with arthritic knees. Wang and Hsu [28] reported the surgical results of medial closed DFO for combined valgus/patellofemoral osteoarthritis with an average follow-up period of 99 months. In their series, 8 of the 30 included knees exhibited a progression of patellofemoral osteoarthritic changes during the follow-up period; however, they reported that these radiographic changes were not correlated with clinical symptoms or functional deficit. We believe that these results are consistent with our study showing that medial closed DFO for valgus deformity does not affect the height, tilt or shift of the patella in patients with arthritic knees.

### Limitations

There are several limitations in the design and content of this study. First, this is a retrospective study. Although the study results represent a single surgeon’s series, there may have been biases in surgical indication and selection. Second, the mean changes in the patellofemoral angles of mTFA were 4.4° preoperatively and −0.2° postoperatively. Because these changes from valgus to varus were small, with a mean change of approximately 4.0°, it is possible that the patellofemoral geometry was not affected. Third, the patellar height was measured on the standing lateral radiograph in an extended position. The Insall–Salvati [14], Caton–Deschamps [15] and Blackburne–Peel indices [16], in their original descriptions, were based on the measurement on the nonweight-bearing lateral radiograph in mild degrees of flexion (30°) [29]. Each parameter is affected by the knee flexion angle at the time of imaging in addition to the presence or absence of loading. Although some reports have stated that the effect of knee flexion angle is not clinically significant [30], we believe that it is important to perform imaging in the same position; thus, in this study, we performed measurements in the extended position. Fourth, only 1- and 2-year results were examined in the analysis of this study. Although our results did not show any substantial changes from 1 to 2 years postoperatively, longer-term results should be investigated for time-dependent changes in clinical and radiological outcomes. Last, there was only one observer in this study.

## Conclusion

In this study, we evaluated the effects of patellar positions on valgus deformity in patients with osteoarthritis of the knee who underwent a medial closed DFO. There were no significant changes in the preoperative, 1-year postoperative or 2-year postoperative patellar height and patellofemoral alignment. Therefore, to highlight the clinical relevance of our findings, medial closed-wedge DFO for the valgus knee does not adversely affect the patellofemoral joint.

## Data Availability

The datasets obtained and/or analyzed during the current study are available from the corresponding author on reasonable request.

## Abbreviations

DFO: Distal femoral osteotomy
mISI: Modified Insall–Salvati Index
mCDI: Modified Caton–Deschamps Index
mBPI: Modified Blackburne–Peel Index
LPT: Lateral patellar tilt
LPS: Lateral patellar shift
mLDFA: Mechanical lateral distal femoral angle
mMPTA: Mechanical medial proximal tibial angle
mTFA: Mechanical tibiofemoral angle
